# Comparison of the Outcomes of Early Versus Late Tracheostomy in the Treatment of Critically Ill Patients: A Retrospective Multicenter Measurement Study Done in Two Hospital Centers in Lebanon

**DOI:** 10.7759/cureus.11361

**Published:** 2020-11-06

**Authors:** Mohamad K Moussa, Ali Moussa, Firas Nasr, Zaynab Khalaf, Safaa Sarout, Nabil Moukarzel, Alfred Dib

**Affiliations:** 1 Orthopedic Surgery, Lebanese University, Faculty of Medical Sciences, Beirut, LBN; 2 Pediatrics, Lebanese University, Faculty of Medical Sciences, Beirut, LBN; 3 Internal Medicine and Geriatrics, Lebanese University, Faculty of Medical Sciences, Beirut, LBN; 4 Endocrinology: Diabetes and Metabolism, Lebanese University, Faculty of Medical Sciences, Beirut, LBN; 5 Otolaryngology: Head and Neck Surgery, Sacre-Coeur Hospital, Beirut, LBN; 6 Critical Care Medicine, Sacre-Coeur Hospital, Beirut, LBN

**Keywords:** critically ill patients, trahceostomy, early tracheostomy, apache-ii score, prolonged mechanical ventilation

## Abstract

Background

Benefits of early tracheostomy (ET) versus late tracheostomy (LT) while treating critically ill patients have been a matter of big debate in the last few years. Several meta-analyses tried to prove the benefits of ET in decreasing the duration of mechanical ventilation (MV), the length of intensive care unit (ICU) stay, and the mortality rates. However, no clear guidelines are available yet. This study will focus on comparing the outcomes of early tracheostomy versus late one.

Methods

This is a retrospective study done in two medical and surgical ICUs at “Sacre-Coeur Hospital” and “Rafik Hariri University Hospital” at Beirut, where we reviewed various files of patients who underwent elective tracheostomy for prolonged MV from January 2015 to June 2016. ET and LT were assumed to be procedures performed respectively before and after 10 days of MV. These two groups were subdivided based on the Acute Physiology and Chronic Health Evaluation II (APACHE II) score calculated in the first 24 hours of ICU admission. Data about short- and long-term mortality, the duration of MV, and the length of ICU stay were collected and compared.

Results

From a total of 45 patients, only 25 patients met the inclusion and exclusion criteria of whom 12 (48%) underwent ET and 13 (52%) patients underwent LT. In patients with APACHE II <25 (6 ET and 6 LT), ET was associated with 50% long-term mortality, 9.6 days mean duration of MV and 23 days mean length of ICU stay compared to 57% (P-value=0.05), 78 days (P-value=0.04) and 79 days (P-value=0.012) of respective parameters in LT groups. In patients with APACHE II >25 (6 ET and 7 LT), ET was associated with 50% long-term mortality, 8.6 days mean duration of MV and 24 days mean length of ICU stay compared to 84%, 105 days, 84 days of respective parameter in LT groups.

Conclusions

Our results are suggestive of the superiority of ET because it was associated with a reduced duration of MV, a decrease in the length of ICU stay, and, most importantly, a lower long-term mortality rate.

## Introduction

Tracheostomy is an operative procedure that creates a surgical airway in the cervical trachea [1]. It is most often performed in patients who have had difficulty weaning off a ventilator [2] where it is sought to facilitate weaning by decreasing the work of breathing in patients with limited reserve, decrease the requirement for sedation, and allow for earlier patient mobilization, feeding, and physical and occupational therapy [3].

Tracheostomy, however, is not devoid of risks. Its application for prolonged duration increases the risk of ventilator-associated pneumonia (VAP) by bypassing and disabling the laryngeal mechanisms promoting the oropharyngeal contamination of the bronchial tree and lung [4]. Some studies have shown that airway colonization, tracheobronchitis, and pneumonia were more significant in patients who had undergone tracheotomy than in intubated patients. Moreover, Georges et al. mentioned that other studies using multivariate analysis showed that tracheotomy was a risk factor for developing nosocomial pneumonia [5]. Other complications may include hemorrhage, stoma infection, subcutaneous emphysema, tracheal stenosis, tracheomalacia, and death [3].

Mechanical ventilation (MV) can also lead to barotrauma of the lungs. The resultant alveolar rupture can lead to pneumothorax, pulmonary interstitial emphysema (PIE), and pneumomediastinum [6]. Despite the fact that it is a common procedure, the optimal time for a tracheostomy in the intensive care unit {ICU} is not clearly defined yet [7].

The term “early tracheostomy” was used by many papers, with different definitions. Most studies and meta-analyses define it as tracheostomy performed within less than 10 days of translaryngeal intubation [8] while Shaw et al. define it as performed within less than seven days [9]. On the other hand, Herrit et al. classified patients as enduring very early tracheostomy (ET) (less than four days), ET within four to 10 days, and late tracheostomy (LT) > 10 days [10].

Most studies suggest that ET is preferential to LT in terms of the length of ICU stay, duration of MV, and hospital cost. Puentes et al. reported that ET allows significant benefits in the reduction of postoperative morbidities, with some overall shorter ICU and hospital stays. These benefits ultimately promote faster patient rehabilitation with reduced healthcare costs [11].

Furthermore, the selection of patients and the timing of the decision for a tracheostomy are subjective, as no reliable tests have been established to predict the need for prolonged ventilation [7].

Many factors are incorporated in taking the decision of this procedure. For instance, the patient’s family and siblings always worry about the complications of such an invasive act and thus hinder taking a decision at the proper time. This suggests that tracheostomy is still socially stigmatized and can intimidate both the patient and the family, especially in Lebanon. The family's understanding and comfort are the most important.

In this study, we are trying to increase evidence about the benefits of early tracheostomy when dealing with critically ill patients by retrospectively studying patients who underwent elective tracheostomy for prolonged MV from January 2015 to June 2016 in two medical and surgical ICUs at “Sacre-Coeur Hospital” and “Rafik Hariri University Hospital” at Beirut.

## Materials and methods

We conducted a retrospective multicentered study by analyzing 45 tracheostomy procedures performed by either the open or the percutaneous technique between January 2015 and June 2016 in two mixed ICUs (including medical and surgical patients) at Rafik Hariri University Hospital and Sacre-Coeur Hospital. Inclusion criteria include male and female subjects, age ＞18 years old, patients who underwent MV with translaryngeal intubation for more than seven days, and patients who are having their first tracheostomy. Exclusion criteria include severe traumatic brain injury, postoperative patients, patients with multiple separate ICU admissions during the same hospital stay, and patients with uncontrolled or hematological malignancy.

The primary endpoints of this study were: (1) short-term mortality (reported mortality within 30 days from doing tracheostomy procedure), and (2) long-term mortality (reported mortality within more than one month to one year from doing a tracheostomy procedure).

The secondary endpoints of this study were the length of ICU stay and duration of MV.

The collected data included the patient’s name, age, gender, cause and duration of MV, and Acute Physiology and Chronic Health Evaluation II (APACHE II) score on admission to the ICU. Medical records were analyzed also for the date of intubation, number of intubations, duration of MV before tracheostomy, date of tracheostomy, date of extubation after tracheostomy, duration of MV post-tracheostomy, and duration of ICU stay.

As a matter of fact, the worst values of temperature, mean arterial pressure (MAP), heart rate, respiratory rate, partial pressure of oxygen (PaO2) and/or A/a gradient, serum HCO3, arterial pH, serum sodium, serum potassium, serum creatinine, hematocrit, and white blood cell (WBC) upon admission to the ICU were gathered. The presence of any acute renal failure, Glasgow Coma Score (GCS) (without sedation), presence of any chronic organ insufficiency, and patient’s age were also noted. Surgical status was also obtained and documented. These data were used to obtain the APACHE II score, which suggested the severity of illness classification and the predicted death percentage of each patient [12]. We assume that patients with ET are those who underwent tracheostomy in ≤10 days of MV while those with LT underwent this procedure in >10 days of MV.

Considering long-term mortality/morbidity, we contacted each patient after one year of his ICU admission investigating his survival either by a phone call or by evidence of new admission to the hospital in the same year.

After completing the data collection process, especially pre-designed data for this study were entered into the Statistical Package of Social Science (SPSS, version 22, IBM Corp., Armonk, NY), which was used for data cleaning and analyses.

Numbers and percentages were used to present categorical variables (mortality, duration of mechanical ventilation, length of ICU stay) while median and range were used to present continuous variables.

In addition, crosstabs were used to cross-tabulate two variables thus displaying their relationship in tabular form. In contrast to frequencies, which summarize information about one variable, crosstabs generate information about bivariate relationships.

Also, the Pearson correlation was used to measure the strength and direction of the association that exists between variables. Statistical significance was indicated at the 0.05 level.

Permission to conduct this study was received from the research committee of both Rafic Hariri University (RHUH) and Sacree-Coeur Hospital. As for the patient’s follow-up after his/her discharge from the hospital, we obtained ethical permission from the patient himself/herself or from his surrogate decision-maker (spouse, adult children, parents, etc) to check if the patient has been dead or lost his/her decision-making capacities.

## Results

Clinical and sociodemographic characteristics

Amongst the 45 patients whose data were collected, 20 patients were excluded. Amongst which, four patients were excluded due to multiple ICU admissions, four others due to traumatic brain injury, 11 due to uncontrolled malignancy, and one other was eliminated because the tracheostomy was done before the ICU admission.

The total number of included patients remaining was 25 patients meeting all our criteria. Those were divided into two groups: the ET group contained 12 patients and the LT group contained 13 patients. In each group, patients could have multiple comorbidities (including chronic organ insufficiency-related organ systems: liver, kidney, cardiovascular, and immune system) and the clinical severity (acute PH changes, acute creatinine changes, WBC, electrolytes disturbances, and so on) at their presentation to the ICU could vary greatly, and this would interfere with the confidence interval of the comparison between the 2 groups. Thus, each group was subdivided into two subgroups based on the APACHE II score, making the comparison more reasonable. The subgroups will be as such: subgroup-A: APACHE II <25 and subgroup-B: APACHE II >25.

**Figure 1:**
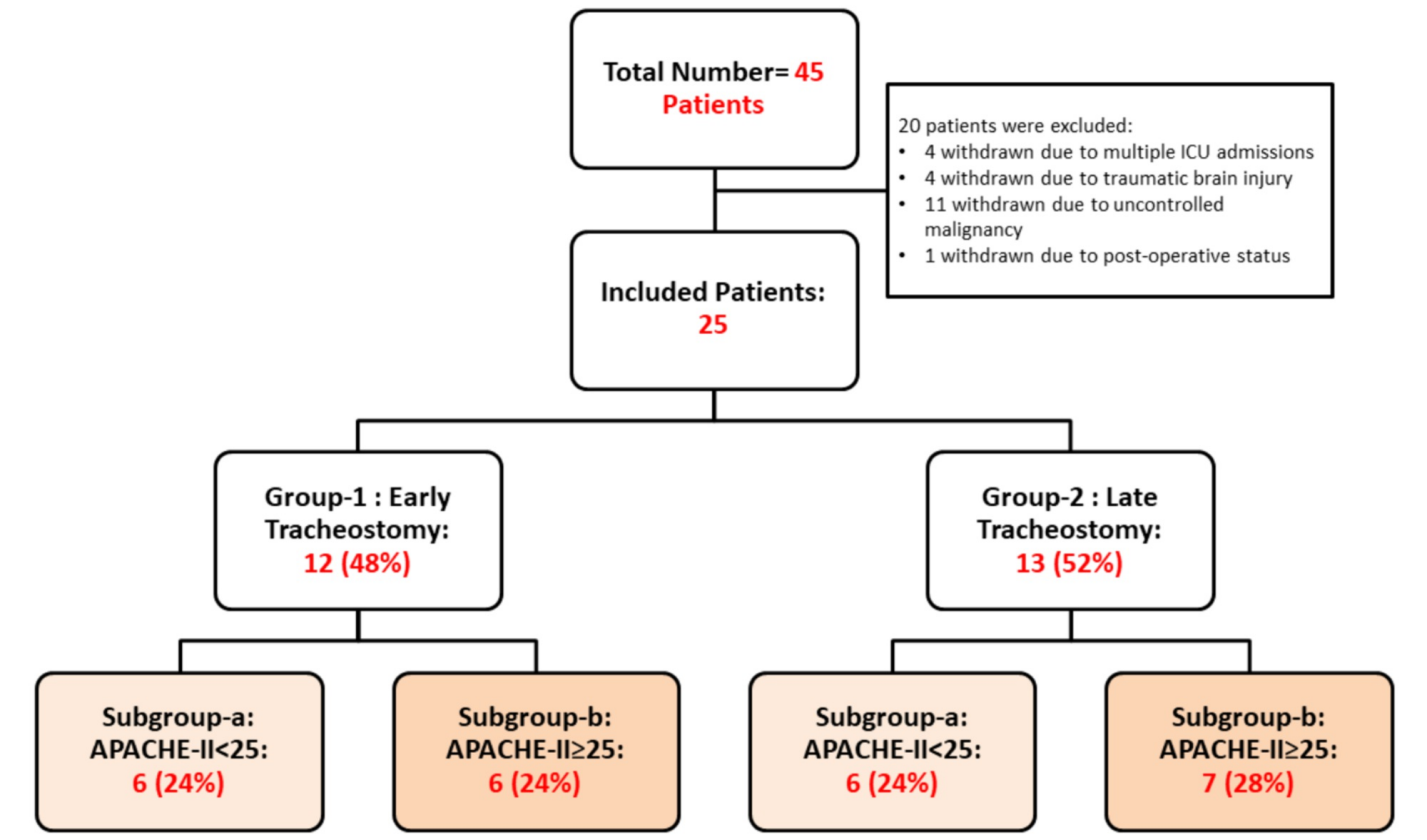
Study design

The study design is shown in Figure 1.

The clinical and socio-demographic characteristics of the population in this study were also collected and compared as shown in Table 1.

**Table 1:** Demographic characteristics of the population in this study COPD: chronic obstructive pulmonary disease; IPF: idiopathic pulmonary fibrosis; CVA: cerebrovascular accident; GCS: Glasgow Coma Scale; ICU: intensive care unit; ARDS: acute respiratory distress syndrome; SAH: subarachnoid hemorrhage; ICH: intracerebral hemorrhage; APACHE II: Acute Physiology and Chronic Health Evaluation II

Characteristics	Early Tracheostomy N=12	Late Tracheostomy N=13
Age (Years)		
Mean	70.5	57
Median	65	58
Male Sex	6 (50%)	7 (53%)
Race		
White	12 (100%)	13 (100%)
Black	0	0
Preexisting Pulmonary Disease (COPD, Asthma, IPF)	6 (50%)	4 (30%)
Preexisting Neurological Disease (CVA, Alzheimer, Dementia, Parkinson)	4 (33%)	6 (46%)
Mean GCS at ICU Admission	9	8
Source of Admission to the ICU		
Emergency Department	10 (83%)	10 (76%)
Hospital Wards	2 (17%)	3 (24%)
Diagnosis at ICU Admission		
Sepsis	4 (33%)	2 (15%)
ARDS	2 (17%)	1 (7%)
Cardiac Arrest	0 (0%)	1 (7%)
Cardiogenic Shock	1 (8%)	2 (15%)
Pneumonia	4 (33%)	4 (30%)
Other Causes (Status Epilepticus, SAH, ICH)	1 (8%)	3(23%)
Number of Intubations Before Tracheostomy		
One Intubation	6 (50%)	10 (76%)
> One Intubation	6 (50%)	3 (24%)
APACHE II Score at Presentation to the ICU		
＜25	6 (50%)	6 (47%)
≥ 25	6 (50%)	7 (53%)

The ET group was slightly older than the LT group, with a mean age of 70.5 years and 57 years, respectively. The two groups were comparable in terms of gender, race, source of admission to the ICU, diagnosis at ICU admission, and number of intubations before tracheostomy.

It was noted that some preexisting pulmonary disease was more common in the ET group while other preexisting neurological conditions were more common in the LT group.

Mortality

**Figure 2:**
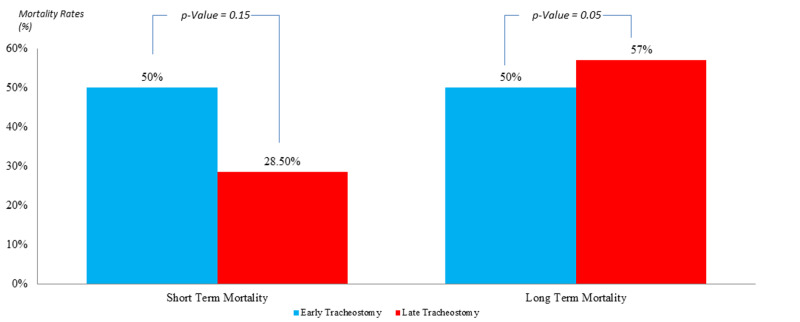
Comparison of mortality rates in patients with APACHE II <25; post early versus late tracheostomy APACHE II: Acute Physiology and Chronic Health Evaluation II

In the population where the APACHE II score is <25 (Figure 2), the short-term mortality rate in the ET group was 50% in comparison to 28.5% in the LT one, with an insignificant P-value of 0.15. However, the long-term mortality rate was 50% in the ET group in comparison to 57% in the LT one, with a statistically significant P-value of 0.05.

**Figure 3:**
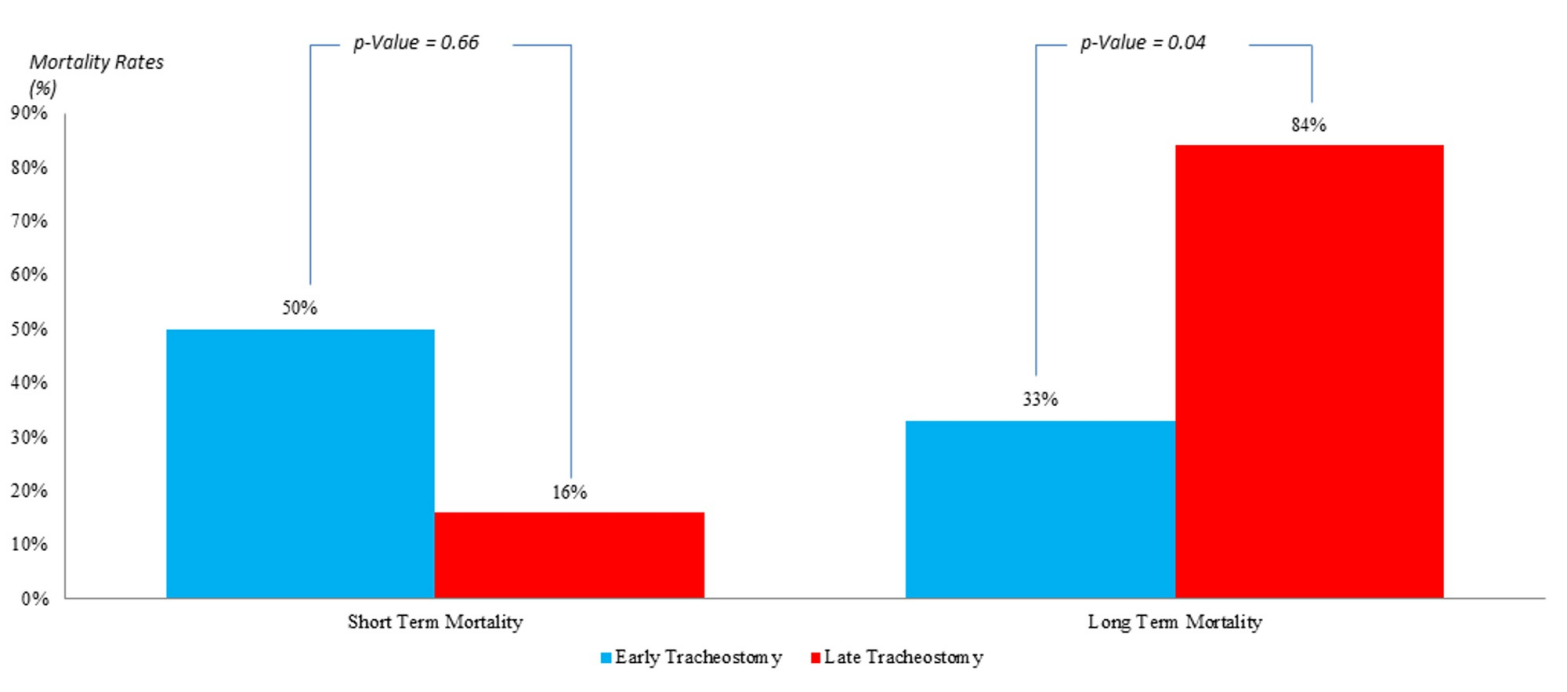
Comparison of mortality rates in patients with APACHE II ≥25; post early versus late tracheostomy APACHE II: Acute Physiology and Chronic Health Evaluation II

In the population where the APACHE II score is >25 (Figure 3), the short-term mortality in the ET group was 50% in comparison to 16% in the LT one with an insignificant P-value of 0.66. However, the level of long-term mortality was 33% in the ET group; a lower value compared to that of the LT one (84%), with a statistically significant P-value of 0.04.

Duration of mechanical ventilation

**Figure 4:**
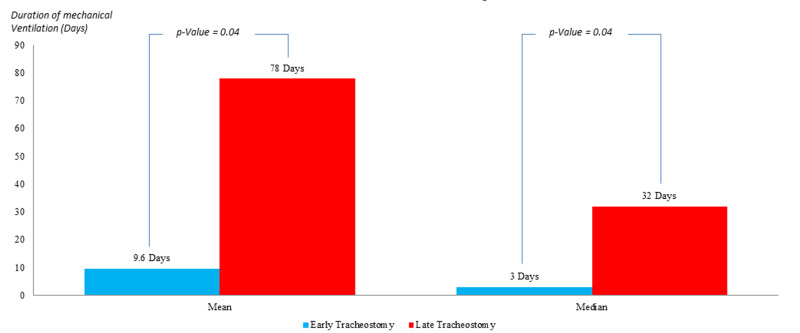
Comparison of the duration of mechanical ventilation in patients with APACHE II<25; post early versus late tracheostomy APACHE II: Acute Physiology and Chronic Health Evaluation II

In the population characterized by an APACHE II score <25 (Figure 4), the mean duration of MV in the ET group was 9.6 days compared to 78 days in the group of LT with a P-value of 0.04, which is statistically significant. The median MV duration in ET patients was three days versus 32 days in the LT group, with a P-value=0.04, which is statistically significant.

**Figure 5:**
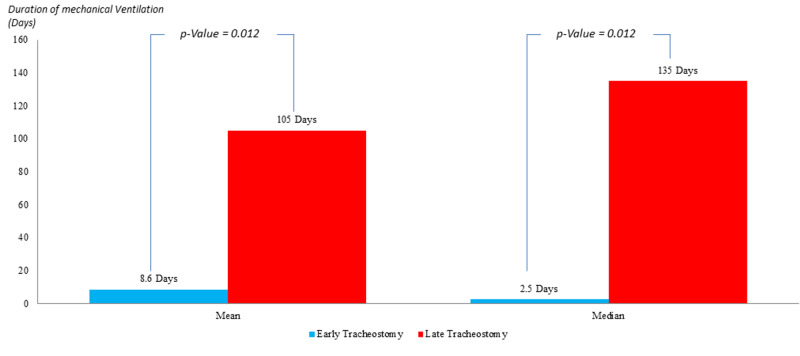
Comparison of the duration of mechanical ventilation in patients with APACHE II >25; post early versus late tracheostomy APACHE II: Acute Physiology and Chronic Health Evaluation II

In the population marked by an APACHE II score >25 (Figure 5), the mean duration of MV in the ET group was 8.6 days as compared to 105 days in the LT group, with a P-value of 0.012, which is statistically significant. The median duration of MV in the ET group was 2.4 days compared to 135 days in the LT group with a P-value of 0.012, which is statistically significant.

Length of ICU stay

**Figure 6:**
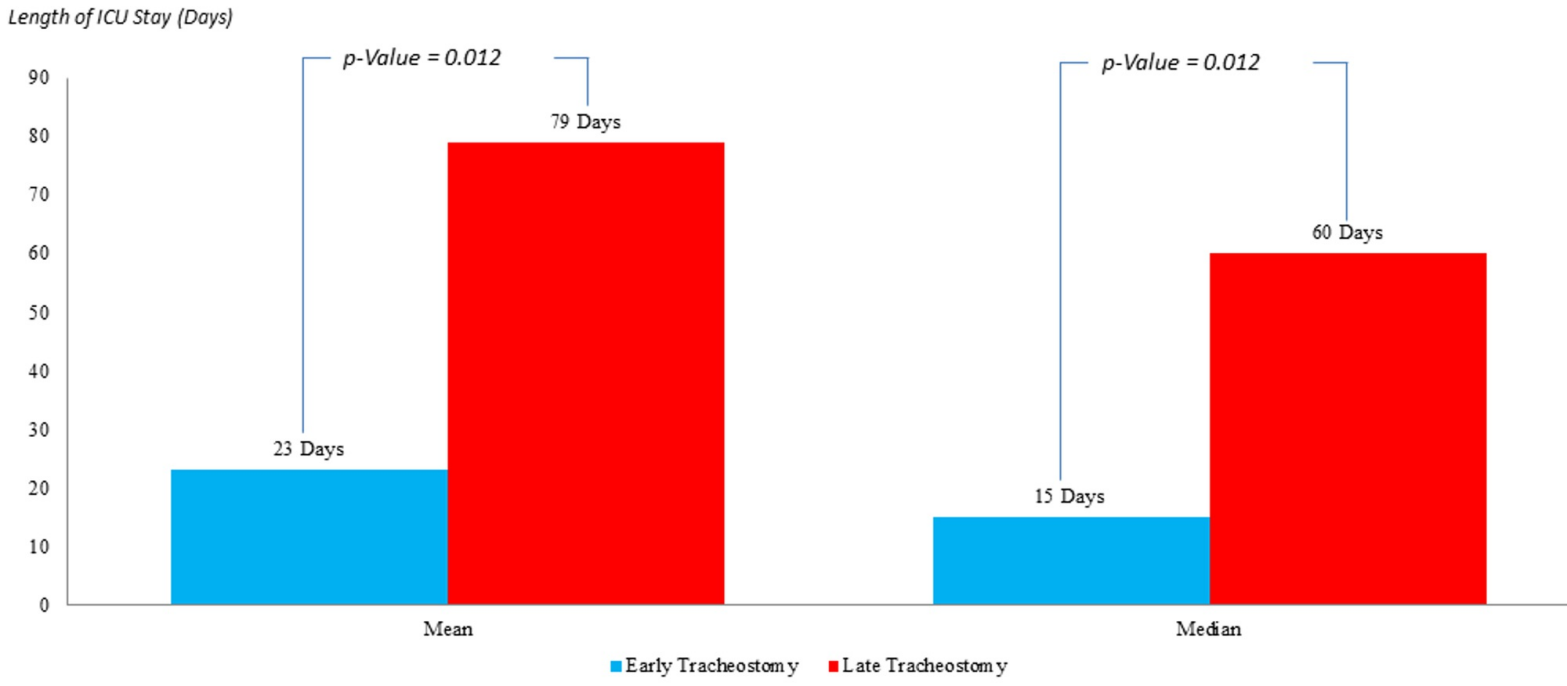
Comparison of the intensive care unit length of stay in patients with APACHE II <25; post early versus late tracheostomy APACHE II: Acute Physiology and Chronic Health Evaluation II

In the population indicating an APACHE II score <25 (Figure 6), the mean length of ICU stay post tracheostomy in the ET group was 23 days as compared to 79 days in the LT group with a P-value of 0.012, which is statistically significant. The median of the ICU stay in ET was 15 days versus 60 days in the LT patients with a P-value of 0.012, which is statistically significant.

**Figure 7:**
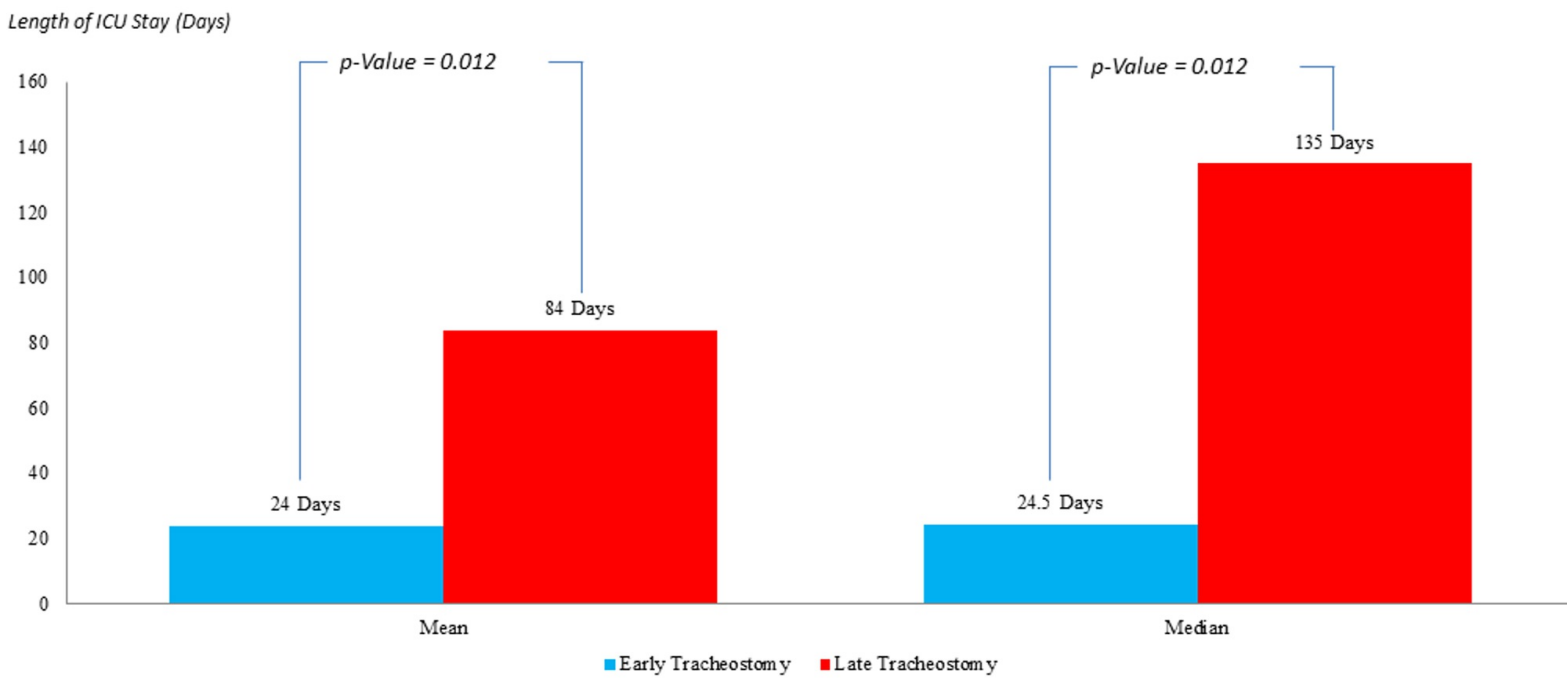
Comparison of the intensive care unit length of stay in patients with APACHE II >25; post early versus late tracheostomy APACHE II: Acute Physiology and Chronic Health Evaluation II

In the population described by an APACHE II score > 25 (Figure 7), the mean length of ICU stay post tracheostomy in the ET group was 24 days as compared to 84 days in the LT group with a P-value of 0.012, which is statistically significant. The median for the length of ICU stay post tracheostomy in the ET group was 24.5 days versus 135 days in the LT group with a P-value of 0.012, which is statistically significant.

Summary of the statistical findings

Amongst patients with APACHE II <25 (6 ET and 6 LT), ET was associated with 50% long-term mortality, 9.6 days mean duration of MV and 23 days mean length of ICU stay as compared to 57% (P-value=0.05), 78 days (P-value=0.04), and 79 days (P-value=0.012), the respective parameters in the LT group (Table 2).

**Table 2:** Summary of the statistical findings in patients with APACHE II < 25 APACHE II: Acute Physiology and Chronic Health Evaluation II

	Early Tracheostomy	Late Tracheostomy
Short-term Mortality	50% (P-value =0.15)	28.5% (P-value =0.15)
Long-term Mortality	50% (P-value =0.05)	57% (P-value =0.05)
Duration of Mechanical Ventilation Post Tracheostomy (days)	Mean = 9.6 Median = 3 (P-value =0.04)	Mean = 78 Median = 32 (P-value =0.04)
Length of ICU Stay Post Tracheostomy (days)	Mean = 23 Median = 15 (P-value =0.012)	Mean = 79 Median = 60( P-value =0.012)

Among patients with APACHE II <25 (6 ET and 7 LT), ET was associated with 33% long-term mortality, 8.6 days mean duration of MV and 24 days mean length of ICU stay compared to 84% (P-value=0.04), 105 days (P-value=0.04), 84 days (P-value=0.012) respective parameters in LT groups (Table 3).

**Table 3:** Summary of the statistical findings in patients with APACHE II >25 APACHE II: Acute Physiology and Chronic Health Evaluation II

	Early Tracheostomy	Late Tracheostomy
Short-term Mortality	50% (P-value =0.66)	16% (P-value =0.66)
Long-term Mortality	33% (P-value =0.04)	84% (P-value =0.04)
Duration of Mechanical Ventilation Post Tracheostomy (days)	Mean = 8.6 Median = 2.5 (P-value =0.04)	Mean =105 Median = 135 (P-value =0.04)
Length of ICU Stay Post Tracheostomy (days)	Mean = 24 Median = 24.5 (P-value =0.012)	Mean = 84 Median = 78 (P-value =0.012)

## Discussion

In patients with APACHE II <25, ET had shown better results concerning long-term mortality where that was decreased by 7% when compared to LT (from 57 to 50%). This result was statistically significant with a P-value of 0.05. On the other hand, ET has also shown better results on long-term mortality in patients with APACHE II score >25 where mortality was decreased by 51% when compared to LT (from 84% to 33%). Those results were also prominent apart from the APACHE II score where ET decreased long-term mortality by 28%.

This indicates that ET is a more suggested and encouraged method for decreasing long-term mortality apart from the APACHE II score, highlighting the fact that it is associated with a more significant decrease in long-term mortality when the APACHE II score >25. As we know so far, this study demonstrated at this level some new facts that differ from other authors’ conclusions.

The first conclusion is that ET improves long-term mortality. A meta-analysis conducted by Huang et al. (2014), evaluating nine studies with 2,072 participants to compare important outcomes between ET and LT, found that no difference in mortality rates was present between patients undergoing early versus late tracheostomy [13]. Also, Meng et al. had the same results when they conducted a metanalysis, including all randomized controlled trial (RCTs) found on Pubmed, EMBASE, and Cochrane from inception to April 2014 and concluded that ET did not significantly alter the mortality, the incidence of VAP, the duration of MV, and the ICU length of stay [14]. Also, in the literature, according to Puentes et al., a recent systematic review of adult ICU patients suggests that ET may reduce the duration of MV and length of ICU stay without any reduction in mortality (11).

The second conclusion is that the APACHE II score can tell which group of patients will benefit more from ET, thus encouraging clinicians to take early accurate decisions when treating critically ill patients: ET decreases long-term mortality by 7% and 53% in patients with APACHE II scores <25 and >25, respectively. The corresponding “number needed to treat” records are 14.2 and 1.9, respectively (Table 4).

**Table 4:** Effect of early versus late tracheostomy on long-term mortality depending on the APACHE II score

	Long-term Mortality in Patients with APACHE II <25	Long-term Mortality in Patients with APACHE II >25
Early Tracheostomy	50%	33%
Late Tracheostomy	57%	84%
Attributable Risk Reduction (%)	7%	51%
Number Needed to Treat (Patients)	14.2	1.9

This means that among every 14 patients having an APACHE II score <25 treated with ET, we can save one life. However, among every two patients having an APACHE II score >25 treated with ET, we would save one life.

These results reintroduced the importance of calculating the APACHE II score in every intubated patient; this fact has been neglected by McHenry et al, who suggested in 2014, “to stop calculating the APACHE II score on all intubated mechanically ventilated patients since there appears to be no clear link between the score and the prediction of need for tracheostomy in this patient population” [15].

On the other hand, our data were statistically insignificant for short-term mortality, whether early or late tracheostomy was performed, with a P-value of 0.15 and 0.66, respectively, and this is compatible with the meta-analysis listed above.

With respect to overall mortality, we noticed that it was similarly high. It was around 93% in both early and late tracheostomy, which could be due to the fact that those patients are critically ill with multiple comorbidities and diseases. Thus, it’s better to stand a while and skim all the all-cause mortality factors in order to avoid any preventable factor hence decreasing overall mortality.

Our data indicate that ET was the preferred method to approach critically ill patients, as it facilitated their weaning from ventilators and decreased the duration of MV. In this way, we theoretically restrict ventilator-associated complications such as infectious complications (nosocomial pneumonia) and hospital-acquired pressure injuries (pneumothorax, pulmonary interstitial emphysema, and pneumo-mediastinum), which increase the cost of the patient’s healthcare and contribute to decreased patient and family satisfaction [6,16].

By analyzing the collected data and the results of our study, we concluded that ET is superior to LT at the level of the length of ICU stay. This is compatible with the conclusion found by Koch et al. who made a study of 100 critically ill patients for over two years and found that ET has the advantages of reducing the time of ventilation and the duration of the ICU stay [17].

Our study is not without limitations; it is retrospective in nature, and this precludes us from drawing any causality between ET and the decrease of postoperative morbidity and mortality. Furthermore, we did not perform a formal cost analysis to confirm the financial benefit of ET on the social scale but, theoretically, reductions in the duration of MV, length of ICU stay, and hospital length of stay are always associated with considerable funds savings and resource optimization. Finally, the number of patients who underwent tracheostomy is quite low when compared to other studies, and this would decrease the significance of our study. At this point, we should mention one of the most important factors limiting the number of patients included (especially in the ET group), which is the family agreement on the procedure as that postpones ET altering it into LT.

For these reasons, we suggest conducting the same study on a larger sample, including more intensive care centers. We also suggest conducting more educational programs for all contributors to the tracheostomy decision, including family, medical staff, and consultants, because interdepartmental consultations in addition to family’s hesitation create a time gap that delays an intervention that could improve the patient’s outcome, mortality, and morbidity.

## Conclusions

In our study, the results are suggestive of the superiority of ET (less than 10 days) in the clinical scenario, where we are obliged to make a decision that affects the life of a critically ill patient. As a matter of fact, this is supposed to be associated with a reduced duration of MV, a decrease in the length of ICU stay, and, most importantly, lower long-term mortality rates. This should be reflected by decreasing the rate of hospital-acquired infections and decreasing healthcare costs.

While this article has no added value regarding the length of ICU stay and MV duration when comparing early versus LT since all studies showed the superiority of ET to LT on these two levels, it is one of the pioneers to demonstrate the superiority of ET to LT with respect to mortality rates, taking into consideration the APACHE II score. Reintroducing the APACHE II score as a scientific criterion that segregates patients at admission to the ICU will allow clinicians to take early accurate ET decisions when the patient is supposed to undergo prolonged MV and will facilitate physician/family agreement on the procedure. The time gap caused by interdepartmental consultation for tracheostomy is due primarily to the absence of clear guidelines about this topic, and this is considered an independent factor for delayed tracheostomy. This study adds further documentation on the impact of early tracheostomy on the patient’s morbidity and mortality that, if added to other similar studies with larger sample size in the future, could be used for establishing international guidelines shortening the time gap and improving the patient’s outcomes, mortality, and morbidity.

## References

[1] Mitchell RB, Hussey HM, Setzen G (2013). Clinical consensus statement: tracheostomy care. Otolaryngol Head Neck Surg.

[2] MacIntyre NR, Cook DJ, Ely EW Jr (2001). Evidence-based guidelines for weaning and discontinuing ventilatory support: a collective task force facilitated by the American College of Chest Physicians; the American Association for Respiratory Care; and the American College of Critical Care Medicine. Chest.

[3] Alhajhusain A, Ali AW, Najmuddin A, Hussain K, Aqeel M, El-Solh AA (2014). Timing of tracheotomy in mechanically ventilated critically ill morbidly obese patients. Crit Care Res Pract.

[4] Ranes JL, Gordon SM, Chen P, Fatica C, Hammel J, Gonzales JP, Arroliga AC (2006). Predictors of long-term mortality in patients with ventilator-associated pneumonia. Am J Med.

[5] Georges H, Leroy O, Guery B, Alfandari S, Beaucaire G (2000). Predisposing factors for nosocomial pneumonia in patients receiving mechanical ventilation and requiring tracheotomy. Chest.

[6] Luo F, Annane D, Orlikowski D, He L, Yang M, Zhou M, Liu GJ (2017). Invasive versus non-invasive ventilation for acute respiratory failure in neuromuscular disease and chest wall disorders. Cochrane Database Syst Rev.

[7] Park YS, Lee J, Lee SM (2012). Factors determining the timing of tracheostomy in medical ICU of a tertiary referral hospital. Tuberc Respir Dis (Seoul).

[8] Keeping A (2016). Early versus late tracheostomy for critically ill patients: a clinical evidence synopsis of a recent Cochrane Review. Can J Respir Ther.

[9] Shaw JJ, Santry HP (2015). Who gets early tracheostomy? Evidence of unequal treatment at 185 academic medical centers. Chest.

[10] Herritt B, Chaudhuri D, Thavorn K, Kubelik D, Kyeremanteng K (2018). Early vs. late tracheostomy in intensive care settings: impact on ICU and hospital costs. J Crit Care.

[11] Puentes W, Jerath A, Djaiani G, Cabrerizo Sanchez R, Wąsowicz M (2016). Early versus late tracheostomy in cardiovascular intensive care patients. Anaesthesiol Intensive Ther.

[12] Knaus WA, Draper EA, Wagner DP, Zimmerman JE (1985). APACHE II: a severity of disease classification system. Crit Care Med.

[13] Huang H, Li Y, Ariani F, Chen X, Lin J (2014). Timing of tracheostomy in critically ill patients: a meta-analysis. PLoS One.

[14] Meng L, Wang C, Li J, Zhang J (2016). Early vs late tracheostomy in critically ill patients: a systematic review and meta-analysis. Clin Respir J.

[15] McHenry KL, Byington RL, Verhovsek ES, Keene S (2014). A study of the relationship between APACHE II scores and the need for a tracheostomy. The Internet Journal of World Health and Societal Politics.

[16] Dixon LM, Mascioli S, Mixell JH, Gillin T, Upchurch CN, Bradley KM (2018). Reducing tracheostomy-related pressure injuries. AACN Adv Crit Care.

[17] Koch T, Hecker B, Hecker A (2012). Early tracheostomy decreases ventilation time but has no impact on mortality of intensive care patients: a randomized study. Langenbecks Arch Surg.

